# Built-In Piezoelectric Nanogenerators Promote Sustainable and Flexible Supercapacitors: A Review

**DOI:** 10.3390/ma16216916

**Published:** 2023-10-27

**Authors:** Shuchang Meng, Ning Wang, Xia Cao

**Affiliations:** 1 Center for Green Innovation, School of Mathematics and Physics, University of Science and Technology Beijing, Beijing 100083, China; 2 Beijing Institute of Nanoenergy and Nanosystems, Chinese Academy of Sciences, Beijing 100083, China

**Keywords:** piezoelectric nanogenerator, built-in, supercapacitors, flexibility, sustainability

## Abstract

Energy storage devices such as supercapacitors (SCs), if equipped with built-in energy harvesters such as piezoelectric nanogenerators, will continuously power wearable electronics and become important enablers of the future Internet of Things. As wearable gadgets become flexible, energy items that can be fabricated with greater compliance will be crucial, and designing them with sustainable and flexible strategies for future use will be important. In this review, flexible supercapacitors designed with built-in nanogenerators, mainly piezoelectric nanogenerators, are discussed in terms of their operational principles, device configuration, and material selection, with a focus on their application in flexible wearable electronics. While the structural design and materials selection are highlighted, the current shortcomings and challenges in the emerging field of nanogenerators that can be integrated into flexible supercapacitors are also discussed to make wearable devices more comfortable and sustainable. We hope this work may provide references, future directions, and new perspectives for the development of electrochemical power sources that can charge themselves by harvesting mechanical energy from the ambient environment.

## 1. Introduction

In 2006, Wang and his colleagues manufactured piezoelectric nanogenerators (PENGs) using ZnO Nanowire (NW) arrays [1]. Notably, PENGs have demonstrated immense potential as a sustainable energy source owing to their capacity to transform mechanical energy into electrical energy [2]. In the past years, PENG has undergone significant improvements in performance, including energy conversion efficiency, cycling stability, and electrical output [3,4,5,6], and PENG-based devices have gone from producing only nano-amps to producing hundreds of volts and hundreds of micro-amps on a considerable scale [7,8,9,10].

PENG has been applied to various applications, including biomedical devices [11,12,13,14], self-powered sensing systems [15,16,17], human–machine interfaces [18,19,20], biomechanical energy harvesting [21,22,23], environmental energy harvesting [24,25,26], and transportation systems [27]. The energy density of PENG has reached over 1KW m^−3^, as indicated in recent research [21]. Following this path, the selection of piezoelectric materials increases its piezoelectric coefficient by changing the composition of the piezoelectric material, and the structure optimization employs techniques such as multilayer structures and nanostructures to increase the surface area and interfacial effects of the piezoelectric material, thus further increasing the power generation efficiency and power density [4,28]. Taking these recent developments into account, PENGs can be considered one of the most significant creative inventions in the field of energy harvesting [29,30].

Meanwhile, flexible and wearable energy-harvesting devices have gained significant attention in industrial and academic communities as they can be integrated with textiles or hand-held portable electronic devices [31]. In recent years, there has been a continuous increase in research on flexible wearable energy devices, focusing mainly on stretchability, flexibility, and mechanical strength [32,33,34,35,36]. As a result, energy storage devices are expected to be compatible with flexible devices so that they can meet the power requirement in applications such as health detection, and electronic skins are also drawing considerable attention [37,38].

Extensive research is currently underway to explore electrochemical energy storage (EES) devices, which include lithium-ion batteries (LIBs) and supercapacitors (SCs), in order to address the increasing need for wearable electronics [39,40]. However, for the long-term use of flexible electronics, such energy storage devices need to be recharged repeatedly, which is determined by intrinsic drawbacks such as limited volume capacity; even the power consumption of wearable devices has been considerably diminished in recent years [41,42,43]. In contrast, self-powered devices combine energy harvesting devices with energy storage devices and can simultaneously collect and store additional energy at any time and release it when needed, thus allowing for the uninterrupted collection, transmutation, storage, and transmission of energy [41,42].

Self-powered devices can be divided into two configurations: (1) the energy harvester is integrated internally into the energy storage device; (2) the energy harvester is connected externally with the energy storage device via an external bridge rectifier [44,45,46,47,48]. Transmission by wire is more efficient, but this reliance on metallic wires sometimes limits the flexibility and safety of the device. At the same time, wireless transmission also faces efficiency issues, where energy transfer decreases dramatically when the distance increases [49]. Hence, researchers have been working on incorporating energy harvesting devices into energy storage devices to increase flexibility while maintaining the transmission efficiency of self-powered devices [50,51,52]. Combining energy harvesters and energy storage devices within a single unit can lead to a considerable reduction in device size, enhancing its flexibility for diverse applications [53]. To meet these demands, various energy harvesting mechanisms have been explored for internally charging integrated energy storage devices, including the triboelectric effect, piezoelectric effect, and thermoelectric effect [54,55].

Recently, self-powered devices based on the piezoelectric effect have been developed by adding piezoelectric materials to SCs or LIBs, allowing them to be charged without any external power source [56,57]. SCs are categorized based on the charge storage mechanism of the electrode material they utilize, leading to classifications like electric double-layer capacitors (EDLCs), pseudocapacitors, and hybrid capacitors [58]. In EDLC, electric energy is stored by ion adsorption/removal. The Faraday redox reaction occurs on the surface of the pseudocapacitor electrode, which allows the pseudocapacitor electrode material to store a greater charge and increases the energy density of the pseudocapacitor, even if the power density of the pseudocapacitor is lower [59]. The charge is stored in hybrid SCs by means of electrostatic and electrochemical reactions [60]. Pseudocapacitors typically utilize electrode materials like metal oxides, metal sulfides, and conducting polymers [61,62,63,64,65]. Carbon-based materials such as activated carbon and carbon nanotubes are widely used in EDLC [66]. The selection of the energy storage device takes into account the design requirement of the device and the electrical output performance [57].

Generally, SCs have a high power density and low energy density compared to batteries [67]. Although self-powered systems consisting of lithium-ion batteries are not suitable for wearable devices due to the toxic and explosive nature of the electrolyte in lithium-ion batteries, a future replacement for lithium ions with other ions in secondary batteries is believed to endow them with a similarly high power density, fast charging capability, superior cycling stability, and safety and reliability [68,69,70,71,72].

Piezoelectric principles in self-charging piezoelectric supercapacitors (SCPSCs) can be achieved by replacing conventional separators with piezoelectric separators [73]. Poly(vinylidene fluoride) (PVDF) and its copolymers are often used as piezoelectric diaphragms for SCPSCs because of their excellent piezoelectric properties [74]. When applied with a voltage, SCs store electrical energy as electrochemical energy and release it when needed. A typical SC consists of two electrodes separated by an insulating separator filled with electrolytes between the electrodes [75,76,77]. When a voltage is applied to the supercapacitor, positive ions move from the electrolyte to the negative electrode, while negative ions move from the electrolyte to the positive electrode. This process creates a double layer of charge at the interface between the electrode and the electrolyte, which stores energy. As more ions move to the electrodes, the charge on the electrodes increases and the voltage across the supercapacitor increases. The charging process is complete when the voltage across the supercapacitor reaches the desired level and the supercapacitor is fully charged [78,79,80]. The piezoelectric potential generated by piezoelectric separators can be used as a power source in SCPSCs. The electrochemical processes during the charging of SCPSCs are similar to those of normal SCs, the main difference being that the piezoelectric separators can be charged by external pressure. As a result, the built-in piezoelectric nanogenerators in flexible supercapacitors can harvest energy from various sources, such as body movements, vibrations, and environmental factors, providing a self-sustained power source for wearable electronics [33,81,82]. However, the energy density of supercapacitors is still relatively low, and the storage capacity is limited, which still poses certain challenges in wide application [83,84,85,86,87,88,89].

**Figure 1:**
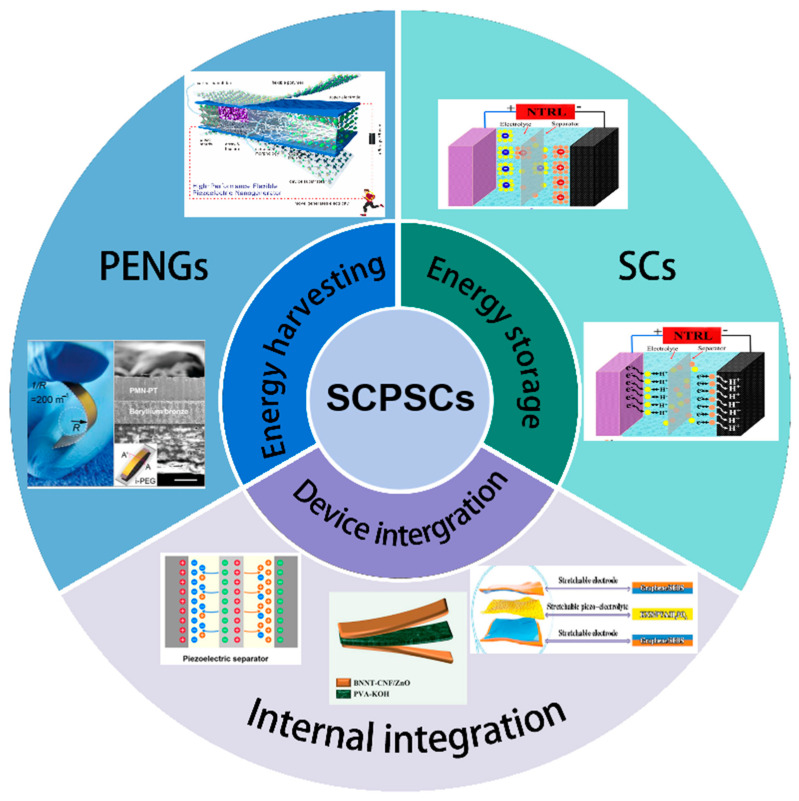
Schematic illustration of the concept and current progress of SCPSCs.

For these reasons, this review article tries to cover the current progress in the working principle, structural configuration, and material selection of various components of SCs with built-in piezoelectric nanogenerators (see Figure 1). As a popular and active research direction, new advances in the integration strategy that lead to the development of self-powered sensors and other multifunctional electronic devices are extensively discussed. While highlighting the adaptative design for various application scenarios, further exploration directions and new challenges in improving the energy storage efficiency and power density of self-charging piezoelectric supercapacitors are also discussed in the hope of providing fundamental insight for researchers to quickly consolidate advances in self-charged SCs.

## 2. Working Mechanism

### 2.1. Working Mechanism of PENGs

PENGs are based on the property of piezoelectric materials, which are easy to be polarized when subjected to external stress, i.e., the piezoelectric effect. The piezoelectric effect is a reversible process that converts external pressure into electrical energy. The strain-induced polarization in the piezoelectric material is due to the mechanical deformation of the lattice. In the initial state, the positive and negative charge centers inside the material; they then coincide, and there is no polarization. When the piezoelectric material is subjected to an external force, the internal positive and negative charges are separated to generate electric dipoles, and the dipole moment changes so that a piezoelectric potential is formed at both ends of the material. Conversely, when an electric field is applied outside the piezoelectric material, the piezoelectric material will generate a corresponding stress. PENG provides a feasible solution for converting mechanical energy into electricity [90]. Piezoelectric nanogenerators were first proposed by Wang and his colleagues in 2006 [1] when they converted nanoscale mechanical energy into electrical energy using ZnO NWs. Deflection aligned NWs using an Atomic Force Microscope (AFM) tip in contact mode. When an NW undergoes bending, it induces stress within the NW, leading to charge separation due to the coupling of its piezoelectric and semiconductor properties. This charge separation, in turn, results in the rectification behavior of the Schottky barrier formed between the metal tip and the NW, ultimately leading to the current generation.

Due to their simple device structure and long-term stability, the application prospect of PENGs is increasingly extensive, including electronic skin, wearable medical devices, and self-powered portable electronic devices [91]. Based on the piezoelectric effect, PENG’s working process can be divided into several steps. Firstly, when an external force is applied to compress or stretch the piezoelectric material within the nanogenerator, it triggers the piezoelectric effect that causes a charge separation phenomenon, resulting in the accumulation of positive and negative charges at opposite ends of the material. Secondly, the electrodes embedded within the nanogenerator are responsible for collecting the separated charges, which generates a potential difference. Thirdly, the potential difference is then converted into a current output through an external circuit. This conversion process enables the conversion of mechanical energy into electrical energy. When the external force is withdrawn, and no external load is present, electrons return to re-establish the charge equilibrium resulting from the reduction of strain. At this time, a current in the opposite direction to the original direction is generated in the external circuit. If a periodic external stress is applied to the PENG to continuously change the internal piezoelectric potential of the material, then the PENG can output a stable pulse current through the external current.

### 2.2. Working Mechanism of Supercapacitors

SCs are energy storage devices that are capable of efficiently storing and discharging large charges. The typical structure of an SC consists of two electrodes with a highly specific surface area, between which a dielectric layer or electrolyte is commonly present to isolate the positive and negative electrodes but allow ions to pass through. The dielectric or electrolyte can be a solid, liquid, or gel-like substance. Electrodes are generally connected to an external circuit for the flow of charge during charging and discharging. These connections may be wires, electrode sheets, or other appropriate conductive materials. SCs are categorized, based on their energy storage mechanisms, into pseudo-capacitors, EDLCs, and hybrid capacitors (asymmetric supercapacitors). EDLCs, in particular, store charge through the physisorption and desorption of electrolytic ions on the electrode surface. This charge storage mechanism is electrostatic (non-Faradaic), meaning no charge transfer occurs between the electrode and the electrolyte, resulting in high reversibility and cycle stability. Whereas pseudocapacitors store charge through fast redox reactions (Faraday process) on/near the electrode surface, asymmetric supercapacitors combine electrostatic adsorption and redox reactions, and the charge storage mechanism of SCs depends on the electrode materials used.

#### 2.2.1. Electric Double-Layer Capacitors

EDLCs harness the process of charge separation on electrode surfaces for energy storage. In the presence of an electrolyte solution between two electrodes, positive and negative charges organize into a double-layer structure on the electrode surfaces, forming what is known as an electric double layer. This electric double-layer has excellent charge storage performance, making electric double-layer supercapacitors characterized by their high power density and long life cycle.

An EDLC comprises two electrodes (usually carbon) and an electrolyte medium. When the capacitor is in an uncharged state, the positive and negative ions in the electrolyte are evenly distributed throughout the capacitor.

**Figure 2:**
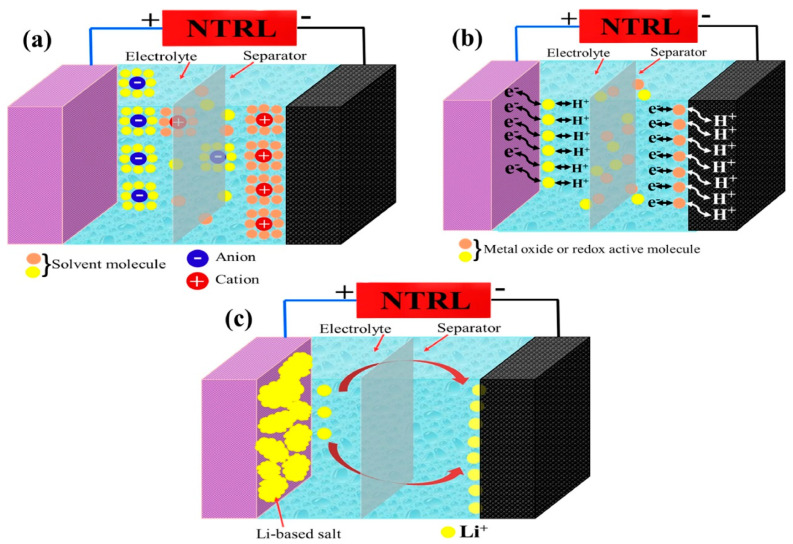
Schematic illustration of different types of supercapacitor technologies. (**a**) electric double-layer capacitor, (**b**) pseudocapacitor, and (**c**) hybrid supercapacitor. Reproduced from ref. [79], with permission. Copyright 2021, Elsevier.

When a voltage is applied to an EDLC, the positive and negative polarized charges attract the corresponding ions in the electrolyte. Positively polarized charges attract anions, forming a tightly packed charge layer, while negatively polarized charges attract cations. This phenomenon results in the formation of two charge-separated regions on the electrode surface, thus creating a double layer of charge. This charge-separated region is the electric double layer, which contains a large charge and can store and release electrical energy in a very short period of time. The energy storage mechanism of an electric double-layer capacitor heavily relies on the process of charge adsorption and desorption in the electrolyte. Figure 2a illustrates the charge storage mechanisms in EDLCs.

#### 2.2.2. Pseudocapacitors

The working mechanism of a pseudocapacitor involves a combination of electrostatic double-layer capacitance (similar to EDLCs) and Faradaic redox reactions. Pseudocapacitors utilize special electrode materials with high surface areas and the ability to undergo fast and reversible redox reactions at the electrode–electrolyte interface. Figure 2b demonstrates the mechanism of the pseudocapacitor.

The pseudocapacitor consists of two electrodes, which are usually made of transition metal oxides, conducting polymers, or other materials with high pseudocapacitance properties. These materials have a large number of active sites on their surfaces, providing a significant surface area for charge storage.

When a voltage is applied across the electrodes immersed in an electrolyte solution, positive ions from the electrolyte are attracted to the negatively charged electrode. In contrast, negative ions are attracted to the positively charged electrode. This attraction leads to the adsorption of positive ions on the negative electrode surface and negative ions on the positive electrode surface, forming an electrical double layer akin to the mechanism in EDLCs.

In the case of pseudocapacitors, additional Faradaic redox reactions occur at the electrode–electrolyte interface. These reactions involve reversible oxidation and reduction processes within the electrode material, entailing electron transfer between the electrode and electrolyte ions. These redox reactions enhance charge storage capabilities beyond the electrostatic double layer, resulting in higher energy densities than pure double-layer capacitors.

During charging, the electrode potential is increased, causing the adsorbed ions to accumulate on the electrode surfaces and the electrode material to undergo redox reactions. This leads to electrical energy being stored in the form of a stored charge. During discharge, the stored charge is released as the ions desorb from the electrode surface and return to the electrolyte solution.

The combination of ion adsorption at the electrode–electrolyte interface and Faradaic redox reactions allows pseudocapacitors to achieve higher energy densities than electric double-layer capacitors while maintaining rapid charge/discharge rates.

#### 2.2.3. Hybrid Capacitors

Hybrid supercapacitors, also known as asymmetric supercapacitors, use different electrode materials for the positive and negative electrodes [92]. One electrode is typically made of activated carbon, which has electrostatic capacitance, while the other electrode is composed of metal oxides or conductive polymers, which have electrochemical capacitance. This mixed configuration allows energy storage through capacitive (electrostatic) and battery-like (Faradaic) processes. The capacitance electrode stores energy by collecting charges on its surface, while the battery-like electrode stores energy through oxidation and reduction reactions involving the insertion or extraction of ions from the electrode material. By combining electrostatic double-layer capacitance and Faradaic pseudocapacitance, hybrid supercapacitors have higher energy storage capacity compared to symmetric supercapacitors. Additionally, due to the larger voltage window of hybrid supercapacitors, they can achieve higher energy density than symmetric supercapacitors [93]. On the other hand, hybrid capacitors fully leverage the advantages of EDLCs and pseudocapacitive electrodes. When voltage is applied, they store electrical energy in two ways during charging. EDLC electrodes accumulate charge by adsorbing ions on their high surface area, while pseudocapacitive electrodes store charge through reversible oxidation and reduction of the electrode material, undergoing Faradaic redox reactions. During the charging of hybrid capacitors, electrical energy is stored in the double layer of the EDLC electrode and also stored through the oxidation and reduction reactions of the pseudocapacitive electrode, as demonstrated in Figure 2c.

### 2.3. Working Mechanism of Self-Charging Piezoelectric Supercapacitors (SCPSCs)

Typically, energy conversion and storage necessitate the utilization of two distinct devices [94]. Most energy harvesting devices suffer from the drawback of low power and voltage output, necessitating the combination of energy harvesters with energy storage devices such as supercapacitors and batteries [95]. Self-charging properties are obtained in SCPSCs by using piezoseparators instead of conventional separators. Piezoelectric materials possess the unique ability to generate potential differences between two surfaces of the material under mechanical stress, such as pressure or vibration. The resulting piezoelectric potential can be used as the power source. The electrochemical reactions that occur during the self-charging of SCPSCs are similar to the charging mechanism of conventional SCs, with the difference that no external power source is required for charging.

**Figure 3:**
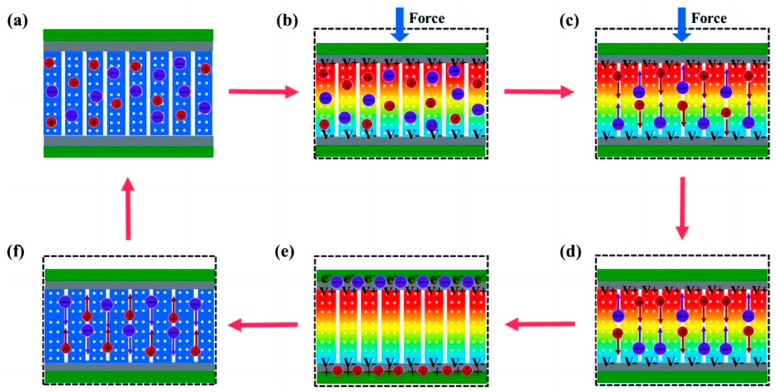
Schematic illustration of the self-charging mechanism of self-charging piezoelectric supercapacitors. (**a**) the initial state without any deformation; (**b**) when an external force is employed on the device, a piezoelectric potential across the separator is generated; (**c**) the generated piezoelectric potential drives positive ions migrating across the separator from the positive electrode to the negative electrode, referred to as the charging process; (**d**) when the external mechanical deformation is removed, piezoelectric potential at the separator is still temporally maintained because the internal residual strain continues to drive the migration of positive ions; (**e**) electrochemical equilibrium is finally obtained; (**f**) piezoelectric potential disappears along with the end of residual strain, leading to a reverse migration of the ions toward the original position, known as self-discharging. Reproduced from ref. [99] along with permission. Copyright 2020, Royal Society of Chemistry.

Within SCPSCs, energy is primarily stored at the electrode–electrolyte interface, and the self-charging mechanism is founded upon electrochemical redox reactions (Faraday reactions) driven by piezoelectric potential [96]. Figure 3 shows the energy conversion and storage process of SCPSCs. As shown in Figure 3a, the device initially exists in a discharged state, devoid of external stimuli or electrochemical reactions at the electrode–electrolyte interface, with the electrode in electrochemical equilibrium with the electrolyte. Upon exposure to an external force, the piezoelectric material generates an internal field, inducing a transient free electron flow and piezoelectric potential, which polarizes the piezoelectric separator (Figure 3b). This ion polarization creates a potential difference between the separator’s extremities, driving electrolyte ions toward the electrodes and causing an electrochemical imbalance.

To reestablish the electrochemical equilibrium, oxidation-reduction reactions (Faraday reactions) transpire on the positive and negative electrode surfaces. Oxidation reactions occur through cation rejection, forming mobile electrons at the positive (cathode) site. In essence, ions migrate to the electrodes via ion-conducting pathways, where redox reactions occur. Electrons generated at the positive electrode traverse toward the negative electrode to maintain charge balance and facilitate charging (Figure 3c).

When the external force is removed, as shown in Figure 3d,e, the residual strain inside the piezoelectric film continues to relax, and the piezoelectric potential within the separator gradually decreases, thus breaking the electrochemical equilibrium of the system. Consequently, some ions migrate in the opposite direction, completing a charging process. When the external deformation disappears completely, the piezoelectric potential vanishes, the electrochemical equilibrium is broken, and the ions return along the original path (Figure 3f), finally reaching electrochemical equilibrium (Figure 3a). As the force is continuously applied to the device, the charging process recurs to establish chemical equilibrium, and such charging cycles repeatedly convert mechanical energy directly into electrochemical energy [97].

SCPSCs hold immense potential for applicability across various fields, including wearable electronics, energy harvesting, and self-powered sensing devices. By capturing and exploiting energy that would otherwise be wasted, these innovative systems offer the prospect of significantly augmenting the efficiency and sustainability of energy infrastructures.

Despite its promise, several challenges should be addressed for practical applications, such as enhancing energy density, the life cycle, and device stability and reducing the self-discharge (SD) property of supercapacitor devices [98]. Nevertheless, the potential advantages of SCPSCs render them a compelling area of exploration within the energy storage domain.

## 3. Current Progress of the PENG-Based Self-Charged System

### 3.1. Structure Configuration of SCPSCs

SCs typically feature a structure comprising two high-surface-area electrodes, with an electrolyte and a separator positioned between them. The SCs that have been reported have rigid, bulky structures such as coins, buttons, cylinders, and prisms and flexible structures such as fibers. Supercapacitors can be classified into different structural based on their specific design, which can be divided into interdigitated [100,101,102], sandwiched [103], and hybrid [82]. The interdigitated supercapacitor has two electrodes, typically made of conductive materials like carbon, metal oxides, or conducting polymers. These electrodes are patterned in an interdigitated or interlocking comb-like structure, where the fingers of one electrode are interleaved with the fingers of the other electrode without touching each other [101,104]. This arrangement increases the surface area for charge storage and reduces the ion diffusion path, improving performance [102,105,106]. The sandwiched supercapacitor, also known as a layered or stacked supercapacitor, consists of several layers of materials that work together to store and release electrical energy.

**Figure 4:**
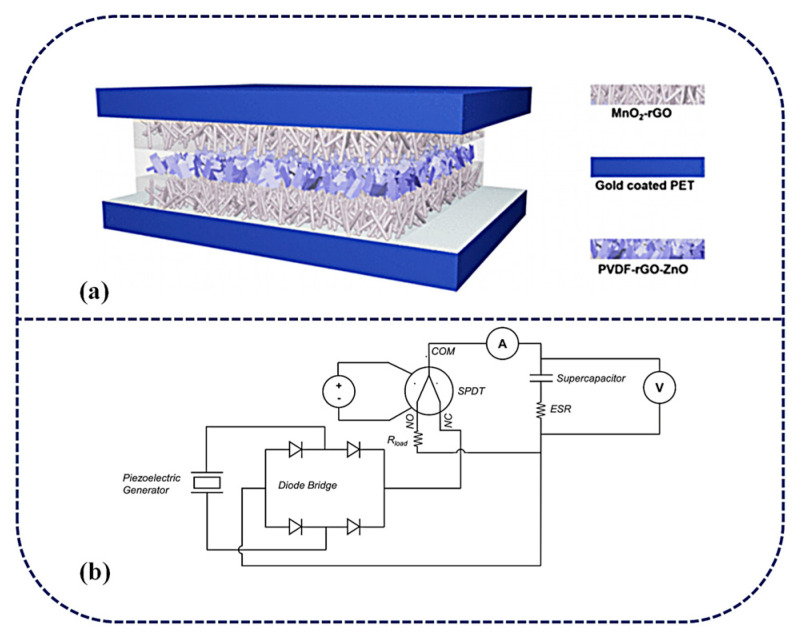
(**a**) Schematic illustration of the as-fabricated, rectification-free, self-charged power unit. Reproduced from ref. [108], with permission. Copyright 2020, American Chemical Society; (**b**) Piezoelectric generator–supercapacitor coupling system. Reproduced from ref. [57], with permission. Copyright 2023, Elsevier.

SCPSCs feature a unique structural design that combines the energy harvesting capabilities of piezoelectric materials with the energy storage capacities of supercapacitors. There are two ways to integrate PENG with SCs: (1) via internal integration (iSCPSC), in which the piezoelectric film in PENG simultaneously acts as a separator for SCs, and (2) the external integration of PENG and SC (eSCPSC), in which separate PENG and SC units are connected by a bridge rectifier and the AC power from the energy harvester is converted to DC power to charge the SC [57,107]. The integrated schematics of iSCPSC and eSCPSC are shown in Figure 4a and b, respectively. Piezoelectric materials as separators, electrolytes, and electrodes to form sandwich-configurated, self-powered supercapacitors are most commonly used in the structure of SCPSCs. For instance, Aamir Rasheed et al. [108] fabricated a piezoelectric film by incorporating ultrathin (<10 nm) zinc oxide nanosheets in polyvinylidene fluoride, which not only acts as a diaphragm but also as a piezoelectric material between SC electrodes. This structure allows self-charging without an external power supply. This design provides a simple and compact structure, making it suitable for various applications.

### 3.2. Material Selection

The successful construction of flexible SCPSCs relies on a meticulous material selection process, emphasizing key factors for optimal performance, flexibility, and durability. Among these factors, the most critical consideration is the material’s piezoelectric properties. The piezoelectric material plays a central role in SCPSCs as it enables the conversion of mechanical energy, such as vibrations or pressure, into electrical energy. Therefore, a high piezoelectric coefficient (d_33_) is imperative for ensuring efficient energy harvesting and storage in the SCPSCs. Piezoelectric materials can be categorized into two main groups: piezoelectric ceramics and piezoelectric polymers. Within the realm of piezoelectric ceramics, there are semiconductor nanomaterials, such as zinc oxide (ZnO) [109,110,111]; lead-based materials like Lead Zirconate Titanate (PZT), PbTiO_3_, and PbZrO_3_ [6,109,112,113]; and lead-free ceramics, including BaTiO_3_, KNN, KNbO_3_, LiNbO_3_, and Na_2_WO_3_.

Piezoelectric ceramics exhibit exceptional mechanical strength, hardness, chemical inertness, and resistance to moisture. Conversely, piezoelectric polymers, like PVDF [6,113] and P(VDF-TrFE) (Poly(vinylidene fluoride-co-trifluoroethylene)), offer distinctive advantages, such as excellent stretchability and biocompatibility. Consequently, they find extensive use in flexible SCPSCs.

#### 3.2.1. Flexible SCPSCs Based on Piezoelectric Electrodes

The piezoelectric electrode, using piezoelectric materials, serves as both the electrode for collecting and storing a charge in supercapacitors and as a means of converting mechanical stress into electrical energy for energy harvesting. In flexible SCPSCs, the piezoelectric electrode should retain a high piezoelectric coefficient while also balancing flexibility and mechanical strength.

In previous research, piezoelectric materials were often integrated into supercapacitors as separators or electrolytes. However, in recent years, there has been a growing interest in utilizing piezoelectric materials as the electrodes in supercapacitors. Lead-free perovskite oxides have shown excellent piezoelectric performance as a pseudo-capacitive material. The charge storage mechanism in lead-free perovskites relies on the rapid redox reactions involving oxygen ions intercalation [114,115]. Due to the absence of a center of symmetry in lead-free perovskite materials, they exhibit substantial spontaneous polarization and a strong piezoelectric response, making them suitable for self-powered piezoelectric supercapacitors.

**Figure 5:**
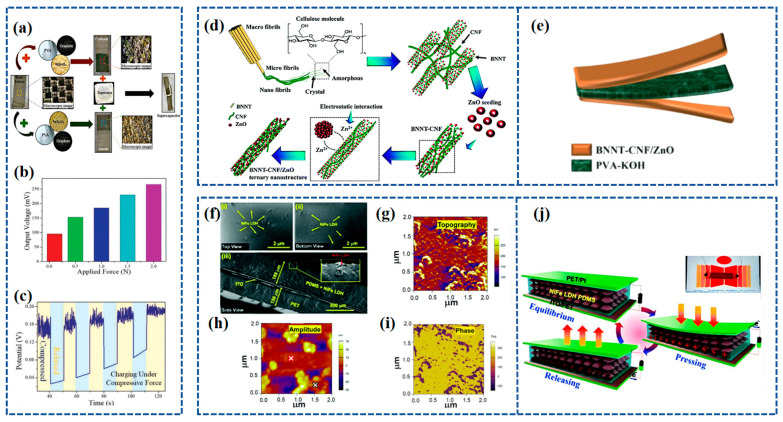
(**a**) Fabrication of SCAPSC; (**b**) variation in output voltage for applied force of different magnitudes; (**c**) cycling performance under compressive force. Reproduced from ref. [83], with permission. Copyright 2022, Wiley; (**d**) schematic illustration of the synthesis mechanism for the BNNT–CNF/ZnO ternary nanostructure; (**e**) schematic illustration of the solid-state flexible device. Reproduced from ref. [116], with permission. Copyright 2022, Royal Society of Chemistry. (**f**) FE-SEM of NiFe LDH:PDMS piezoelectric composite films in (i) top-view, (ii) top-view, and (iii) side-view [117]; (**g**) topography of NiFe LDH in a 2 mm scanning region using PFM [117]; (**h**) amplitude map of NiFe LDH in a 2 mm scan region using PFM [117]; (**i**) phase diagram of NiFe LDH in a 2 mm scanning region using PFM [117]; (**j**) schematic of the working of the NiFe LDH nanogenerator and (inset) real image of the NiFe LDH device. Reproduced from ref. [117], with permission. Copyright 2022, Royal Society of Chemistry.

Recently, Bhavya Padha et al. [83] fabricated a fully solid-state Self-Charging Asymmetric Piezoelectric Supercapacitor (SCAPSC) using nickel stannate (NiSnO_3_) as the positive electrode, iron stannate (FeSnO_3_) as the negative electrode, and Polyvinyl Alcohol–Potassium Hydroxide (PVA-KOH) thin film as the ion gel electrolyte, as shown in Figure 5a. The specific capacitance of FeSnO_3_ reached 2853 F g^−1^, which was the highest among the perovskite-based electrodes. According to their experimental results, the self-charging phenomenon achieved a maximum voltage increase from 91 mV to 266 mV when applying a continuously increasing force from 1 N to 20 N (Figure 5b). The prepared perovskite supercapacitor demonstrated high flexibility, robustness, stability, excellent electrochemical performance, and self-charging potential. The device was charged under a constant compressive force, as shown in Figure 5c, with the voltage steadily increasing during the compression process and returning to the initial stage when the applied force was removed.

ZnO crystals possess a non-centrosymmetric crystal structure, which gives rise to its piezoelectric properties. When subjected to mechanical stress or pressure, the crystal lattice deforms, resulting in the separation of positive and negative charges along different crystallographic axes, creating an electric dipole moment. Iqra Rabani et al. [116] synthesized ZnO nanoparticles (NPs) on the surface of Cellulose nanofibers (CNFs) and BNNT using a hydrothermal method. The synthesis process of BNNT-CNF/ZnO is depicted in Figure 5d. The outcome yielded a notable ternary nanostructure comprising BNNT-CNF/ZnO, exhibiting a specific capacitance of 300 F g^−1^ alongside a high energy density of 37.5 W h kg^−1^ and a power density of 0.9 kW kg^−1^ at a current density of 1 A g^−1^. The effective piezoelectric coefficient (d_33_) of a 90 μm thick BNNT-CNF/ZnO paper-based device was tested and found to be 12.6 pC N^−1^. Through the incorporation of a PVA-KOH gel electrolyte between the BNNT-CNF/ZnO electrodes, an ultra-thin flexible solid-state symmetric supercapacitor was successfully constructed. This supercapacitor exhibited a remarkable specific capacitance of 94 F g^−1^ at a current density of 1 A g^−1^, demonstrating notable cycling stability with a 97% retention over 5000 cycles and an impressive energy density of 30.3 W h kg^−1^ at a power density of 1.3 kW kg^−1^. The construction of this solid-state flexible symmetric supercapacitor is shown in Figure 5e.

Sushmitha Veeralingam et al. [117] created self-sustained units by combining piezoelectric nanogenerators with high-performance asymmetric supercapacitors (ASCs), employing nickel–iron layered double hydroxide (NiFe LDH)-based piezoelectric nanomaterials that are free from lead. The morphology of the NiFe LDH piezoelectric composite layer, applied to the ITO/PET substrate, was examined through FE-SEM, and the resulting micrographs can be found in Figure 5f. The top-view images, Figure 5f(i) and Figure 5f(ii), reveal the uniform distribution of NiFe LDH nanoparticles within the PDMS matrix. A cross-sectional view of the NiFe LDH:PDMS/ITO-coated PET structure, depicted in Figure 5f(iii), was also obtained. The piezoelectric charge coefficient (d_33_) of NiFe LDH was examined through AFM, resulting in a value of 274 pm V^−1^. The topography of NiFe LDH was observed across a 2 mm scanning area using PFM, as depicted in Figure 5g. The amplitude (Figure 5h) and phase diagrams (Figure 5i) of NiFe LDH were investigated, revealing a two-dimensional nanosheet-like morphology with an average thickness of 20 nm. The actual image of the NiFe LDH structure is presented in Figure 5j. The operation of the nanogenerator is elucidated by the charge generation mechanism depicted in Figure 5j, where the generation of electricity is attributed to the piezoelectricity and capacitance of the NiFe LDH composite film.

#### 3.2.2. Flexible SCPSCs Based on Gel Polymer Electrolyte (GPE)

**Figure 6:**
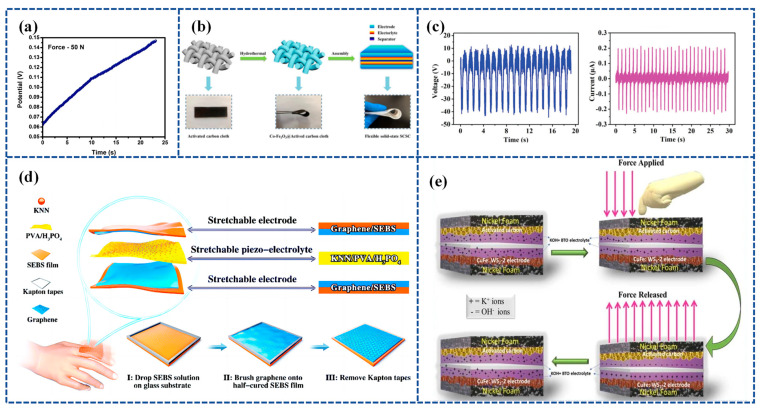
(**a**) Self-charging under the compressive force of 50 N. Reproduced from ref. [118], with permission. Copyright 2022, Elsevier; (**b**) schematic illustration of the synthesis of Co-Fe_2_O_3_@ACC electrode and architecture of flexible solid-state SCSC. Reproduced from ref. [119], with permission. Copyright 2021, Elsevier; (**c**) open-circuit voltage and short-circuit current generated in the KNN/PVA/H3PO4 piezo-electrolyte film; (**d**) schematic illustration of the as-fabricated stretchable SCSC consisting of stretchable graphene/SEBS electrodes and stretchable KNN/PVA/H_3_PO_4_ piezo-electrolytes, and the fabrication process of the stretchable graphene/SEBS electrodes. Reproduced from ref. [99], with permission. Copyright 2020, Royal Society of Chemistry; (**e**) schematic of self-charging and -discharging mechanism in WS_2_ @CuFe-2//AC device. Reproduced from ref. [120], with permission. Copyright 2023, Elsevier.

GPE is a type of electrolyte that is becoming increasingly popular in flexible Self-charging Supercapacitor Power Cells (SCSPCs). Polyvinyl Alcohol (PVA) is a commonly used gel polymer electrolyte in SCs; however, it does not possess piezoelectric characteristics on its own. In order to complete the conversion of mechanical to electrical energy via PVA-based polymer electrolytes, a novel Polyvinyl Alcohol–Potassium Hydroxide Titanate–barium titanate (PVA-KOH-BTO) multifunctional piezoelectric electrolyte nanogenerator that converts mechanical energy into electrical energy is needed [118]. An integrated Self-charging Hybrid Supercapacitor (SCHSC) with a specific capacity of 280 C/g was prepared using a two-dimensional bismuthane–hexagonal boron nitride nanocomposite (Biene-h-BN NC) as the cathode, activated carbon (AC) as the anode, and PVA-KOH-BTO as the piezoelectric electrolyte at 1 A/g. In addition, SCHSC provided a high energy density of 87.5 Wh/kg and a high power density of 11250 W/kg with a maximum self-charging potential of 146 mV under an applied cyclic compression force of 50 N in 23 s (Figure 6a). The prepared SCHSC was capable of self-charging and discharging for eight consecutive cycles under the action of a thumb-impact external force, and this study can be used as a potential candidate for the manufacture of future smart electronic device materials.

Further, Zhou et al. [119] employed co-doped Fe_2_O_3_ deposited on activated carbon cloth (Co-Fe_2_O_3_@ACC) as a sophisticated symmetrical electrode. They also utilized BaTiO_3_ piezoelectric particles combined with a PVA-KCl gel film (PVA-KCl-BaTiO_3_) as a piezoelectric electrolyte. The device has the combined advantages of high flexibility and good electrochemical and self-charging properties. Specifically, it can harvest and store energy concurrently through the force–electric conversion of the piezoelectric effect and is capable of self-charging up to approximately 120 mV with a simple shock, which is achieved through repeated bending at a frequency of 1.0 Hz for 7 min. The device structure is shown in Figure 6b.

Zhou et al. [99] prepared a piezoelectric electrolyte by synthesizing potassium sodium niobate (KNN) mixed with PVA and H_3_PO_4_, referred to as KNN/PVA/H_3_PO_4_. Figure 6c illustrates the output voltage and current of the PENG based on the KNN/PVA/H_3_PO_4_ piezoelectric electrolyte operating at a frequency of 1 Hz. By placing the KNN/PVA/H_3_PO_4_-based piezoelectric electrolyte between graphene-coated elastomeric styrene–butadiene–styrene–benzene electrodes, a highly stretchable and Self-chargeable Supercapacitor (SCSC) device was prepared (Figure 6d). The highly stretchable device, once fabricated, can be charged to around 1.0 V through either pure hand tapping for 300 s at a frequency of 2 Hz or by repeated stretching for 40 s at 1 Hz.

Akshaya Subhramaniyan Rasappan et al. [120] constructed WS_2_ @CuFe nanobox composites using the Kirkendall effect, used WS_2_ @CuFe-2 nanoboxes as electrodes, and used KOH-BTO-PVA piezoelectric electrolytes to construct a WS2 @CuFe-2//AC asymmetric supercapacitor device (SCHSD) when an external force was applied to the SCHSD. This triggered a Faraday reaction at the electrode/electrolyte interface (WS_2_ @CuFe-2 and KOH-BTO) due to the piezoelectrochemical effects, leading to ion polarization. A schematic depicting the self-charging and discharging mechanism in the WS_2_ @CuFe-2//AC device is presented in Figure 6e.

#### 3.2.3. Flexible SCPSCs Based on Piezoelectric Polymer Separators

Materials used as piezoelectric separators should have high dielectric coefficients and high permittivity to improve energy storage capacity ($E=\frac{1}{2}CV^{2}$), therefore increasing the permittivity of the material while maintaining the high voltage coefficient, which is essential for the use of piezoelectric materials in piezoelectric energy harvesters [89]. Piezoelectric ceramic materials (e.g., PZT) have one order of magnitude higher piezoelectric coefficient compared to piezoelectric polymers (d_33_ = 200–600 pC N^−1^), as well as higher electromechanical conversion efficiency. However, the high rigidity of piezoelectric ceramics hinders their application as flexible SCPSC separators.

1. PVDF

PVDF is widely used in SCPSCs due to its excellent piezoelectric properties (d_33_ = 20–25 pC N^−1^) and mechanical stability and can be used in flexible energy harvesting devices due to its strong electrical activity, good durability, light weight, and good biocompatibility. PVDF is frequently employed as a separator due to its chemical stability and inertness across a broad potential range, making it non-reactive with the electrolyte. Compared with other organic polymers, PVDF has superior piezoelectric properties, can be deformed without cracks, and has high breakdown strength [96,121]. As a common piezoelectric material, PZT has one order of magnitude higher piezoelectric coefficient than PVDF and its copolymers. The piezoelectric coefficient of PVDF and its copolymers is about 20~40 Pc/N. However, PZT has a high lead content, which reduces the biocompatibility of PZT.

**Figure 7:**
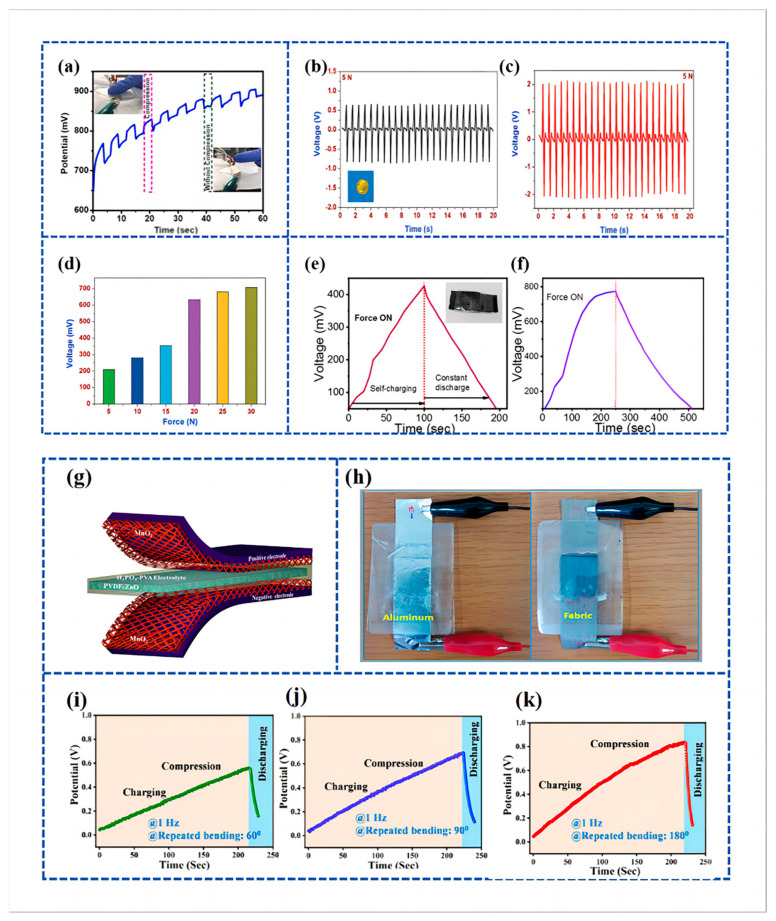
(**a**) Cycling performance and voltage output of under-applied compressive force by finger pressing; reproduced from ref. [122], with permission. Copyright 2023, Elsevier; (**b**,**c**) piezoelectric characterization of the electrospun PVDF and NaNbO_3_/PVDF nanofibrous separator; (**b**) open-circuit voltage profile of the bare PVDF nanogenerator under a compressive force of 5 N. Inset (**b**) shows the digital micrograph of the piezoelectric nanogenerator using NaNbO_3_/PVDF nanofibrous separator with aluminum as the top and bottom electrode; (**c**) open-circuit voltage of the electrospun NaNbO_3_/PVDF nanofibrous separator under compressive force of 5 N; (**d**) comparison of the charging voltage of the MoSe_2_ SCSPC device under various applied compressive forces. Reproduced from ref. [125], with permission. Copyright 2018, Wiley; (**e**,**f**) self-charging behavior of PVDF/CH graphene-based PSC measured two different times with compressive force of 10 N. Reproduced from ref. [13], with permission. Copyright 2022, Elsevier; (**g**) schematic diagram of the fabricated SCSPC. MnO_2_ on aluminum foil is used as the positive and negative electrodes and PVDF-ZnO film as a separator; (**h**) digital images of SCSPC based on aluminum foil and conductive fabric. Reproduced from ref. [97], with permission. Copyright 2015, American Chemical Society; (**i**–**k**) self-charging performance of PSCFS under different bending states, viz., ~60, ~90, and ~180° and discharging through the external load. Reproduced from ref. [126], with permission. Copyright 2023, American Chemical Society.

Amrita De Adhikari et al. [122] applied WS_2_ nanospheres adorned with Polypyrrole (PPy) NWsto supercapacitors and assembled Self-Powered Symmetrical Supercapacitor Devices (SPSSC) using WS_2_@ PPy heterostructured electrode materials and PVDF film separators as piezoelectric generators. The specific capacitance of the electroactive WS_2_@ PPy heterostructure was 245 F g^−1^ at a current density of 1A g^−1^, with a cycling stability of 99.2%. The self-charging piezoelectric effect in the SPSSC device was assessed through finger-pressing, achieving a peak output potential of 880 mV within 60 s. The device exhibited a specific capacitance of 337.73 F g^−1^, an energy density of 26.38 Wh kg^−1^, and a power density of 1874 W kg^−1^, all maintained across a broad voltage range of 1.5 V. The synergistic effect of Polypyrrole and WS_2_ in this work was the main reason for the electrochemical enhancement. Figure 7a showcases the repetitive self-charging behavior of the SPSSC device over 10 successive cycles, confirming its electromechanical stability. The main reason for the enhanced performance is the synergy between Polypyrrole and WS_2_ in this work.

However, the energy conversion efficiency of bare PVDF is slightly lower than that of polarized PVDF or chemically modified PVDF [121,123]. Enhancing the piezoelectric coefficients of PVDF and its copolymers is, therefore, important for the fabrication of flexible piezoelectric SCs. Of the five known phases of PVDF, the β-phase has excellent piezoelectricity, and the α-phase is the most common phase, so improving the formation of the β-phase and the transition from the α-phase to the β-phase is a very important goal. The commonly used methods for treating PVDF are the addition of filler materials, etc. The addition of filler materials is low-cost, easy to manufacture, and can effectively enhance the electrical output of PVDF [124].

**Figure 8:**
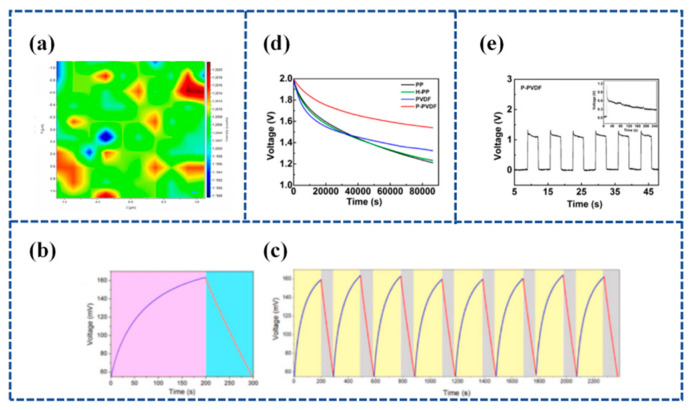
(**a**) represents the intensity ratio map of (IW-O/Iβ) of the PTA-PVDF piezo-polymer electrolyte film; (**b**) self-charging performance of graphene SCSPC charged via external applied force followed by discharging using a constant current; (**c**) repetitive self-charging cycles of graphene SCSPC using PTA-PVDF piezo-solid electrolyte separator. Reproduced from ref. [127], with permission. Copyright 2021, Elsevier; (**d**) *V_oc_* decays; (**e**) voltage difference of the P-PVDF separator under the applied pressure cycle. Reproduced from ref. [74], with permission. Copyright 2021, American Chemical Society.

Pazhamalai et al. [125] employed 2D molybdenum diselenide (MoSe_2_) as the energy storage electrode, while a combination of polyvinylidene fluoride-co-hexafluoropropylene/tetraethylammonium tetrafluoroborate (PVDF-co-HFP/TEABF_4_) ion gel served as the electrolyte. Additionally, polyvinylidene fluoride/sodium niobate (PVDF/NaNbO_3_) nanofibers, prepared using electrostatic spinning, were utilized as the piezoelectric separator in the fabrication of the SCSPC device. The porous mesh structure of the nanofiber piezoelectric separator, the intercalated MoSe_2_ energy storage electrode, and the use of ionic gel electrolyte improved the performance of the fabricated SCSPC. Figure 7b,c provides a comparison of the piezoelectric characteristics between bare PVDF and NaNbO_3_/PVDF when subjected to a cyclic force of 5 N. Under a 5 N force, the peak open-circuit voltage (V_oc_) for the bare PVDF nanofiber separator is approximately 1.2 V, as shown in Figure 7b. Meanwhile, Figure 8c illustrates the piezoelectric response of electrospun NaNbO_3_/PVDF nanofibers, highlighting a significant enhancement in piezoelectric output in comparison to bare PVDF. The peak voltage of the NaNbO_3_/PVDF nanofiber reaches approximately 4 V. The enhancement in the piezoelectric response of the composite can be ascribed to the inclusion of crystalline NaNbO_3_ within the piezoelectric polymer fiber. The fabricated SCSPC device can convert external compressive forces into electrical energy and store it inside the device, which can be charged up to 708 mV within 100 s at a compressive force of 30 N (Figure 7d). The specific capacitance of the device at a constant discharge current of 0.5 mA and a specific power density of 268.91 µW cm^−2^ is 18.93 mF cm ^−2^ with a specific energy of 37.90 mJ cm^−2^.

Sahu et al. [13] used coconut shell (CH) as a filler in the PVDF matrix to effectively improve the piezoelectric performance of PVDF. The piezoelectric supercapacitor was made by using graphene as the electrode and PVDF doped with CH powder as the piezoelectric separator. When a consistent compression force of 10 N was applied at a frequency of 2 Hz, the voltage across the PVDF/CH PSC cell progressively rose from 50 mV to 423 mV over a span of 100 s, as shown in Figure 7e. The device exhibited a notable increase in voltage, surging from 90 mV to 773 mV within 250 s, as indicated in Figure 7f. The maximum energy density of the piezoelectric supercapacitor made was 76.33 mJ/cm^2^, and the power density was 1.316 mW/cm^2^, which was significantly higher than that of other graphene/PVDF-based devices.

Ramadoss et al. [97] engineered a piezoelectrically driven SCSPC. This device utilized MnO_2_ NWs for both positive and negative electrodes (SC), polyvinylidene fluoride-zinc oxide (PVDF-ZnO) films as separators (piezoelectric nanogenerators), and a PVA/H_3_PO_4_ electrolyte, as illustrated in Figure 7g. This system is designed to capture mechanical energy, convert it into electrochemical energy, and subsequently store the converted electrochemical energy. The SCSPC comprises a nanogenerator, a supercapacitor (SC), and a power management system, making it a direct power source. The self-charging capability of the SCSPC was showcased through mechanical deformation caused by the impact of a human hand. The SCSPC (aluminum foil-based) can be charged to 110 mV in 300 s. Further, Figure 7h displays the visual representations of the created SCSPC, which includes aluminum foil and fabric.

Suvankar Mondal et al. [126] fabricated a piezoelectric nanogenerator using different wt % of copper cobalt nickel oxide (CuCoNiO_4_) combined with PVDF and used it as a piezoelectric separator to fabricate a Piezoelectric Self-charging Flexible Supercapacitor (PSCFS). The fabricated piezoelectric nanogenerator exhibited the highest electroactive phase (>86%) at 1 wt % of CuCoNiO_4_ in combination with PVDF, which is referred to as PNCU 1. The PSCFS was assembled utilizing PNCU 1 as a separator, with CuCoNiO_4_ NWs on carbon cloth (CC) serving as both positive and negative electrodes. This involved the use of PNCU 1 as a separator and CuCoNiO_4_ nanowires on CC for the positive and negative electrodes. The devices’ performance was evaluated at bending angles of approximately 60° and 90°, with frequencies set at 1 Hz, resulting in voltage increases to 535 mV and 666 mV, respectively (Figure 7i,j). Subsequently, following 220 s of periodic 180° bending at a frequency of 1 Hz, the open-circuit voltage of the PSCFS device rose from an initial 35 mV to reach a level of 845 mV (Figure 7k).

Manoharan et al. [127] developed a piezo-electrolyte film that included a solid proton-conducting electrolyte, Phosphotungstic Acid (PTA), embedded within the piezoelectric PVDF matrix. This film was utilized as a separator and electrolyte in SCSPC devices. PTA is widely recognized as an excellent electrolyte for supercapacitors, whether in liquid or solid gel form. Various physico-chemical characterizations of the prepared PTA-PVDF piezo-electrolyte film, including X-ray Diffraction (XRD); morphological, elemental, Raman spectroscopy; and mapping analyses (as shown in Figure 8a), indicated a uniform distribution of the PTA-PVDF electrolyte within the PTA-PVDF matrix. The incorporation of PTA into PVDF serves to enhance the performance of mechanical energy-to-electrical energy conversion and the supply of electrolyte ions. A graphene SCSPC device was constructed using the PTA-PVDF piezo-electrolyte film, featuring an area capacitance of 184.94 mF cm^−2^ and an energy density of 59.18 mJ cm^−2^. Under a 2 N compression force, the graphene SCSPC displayed a charging curve with voltage levels ranging from 52 to 162 mV over a span of 200 s (Figure 8b). As shown in Figure 8c, the graphene SCSPC device was discharged using a current of 0.5 mA, and the output of the graphene SCSPC was almost similar for the repeated self-charging process in several consecutive cycles, thus highlighting its excellent electromechanical stability.

In reducing the self-discharge behavior of SCs, Zhao et al. [74] used porous PVDF membranes as supercapacitor separators and prepared PVDF membranes via the polarization-induced piezoelectric method to reduce the self-discharge of SCs. Self-discharge experiments showed that compared with commercial PP separators, the open-circuit voltage (*V_oc_*) decay rate of SCs with this piezoelectric PVDF (P-PVDF) separator (Figure 8d). The reduction in the self-discharge of SCs is due to the fact that the polarized PVDF separator can hinder the movement of ions in both directions, resulting in a slower ion diffusion rate, and the self-discharge caused by the diffusion of electrolyte ions is effectively reduced. The voltage variation of the separator under the applied pressure cycle is shown in Figure 8e [74].

2. P(VDF-TrFE)

**Figure 9:**
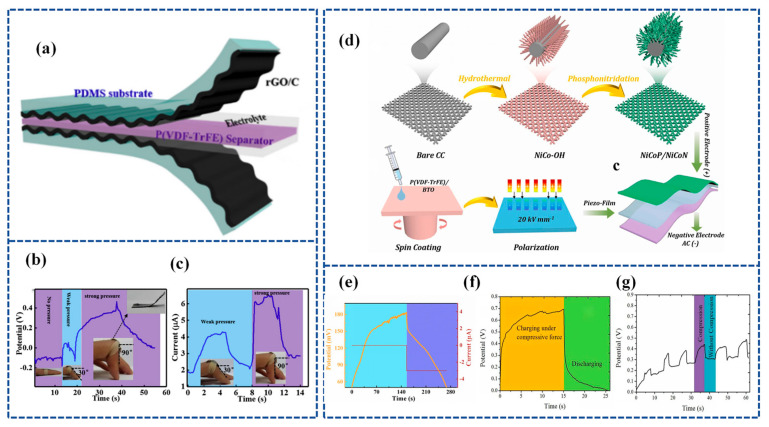
(**a**) The schematic of the fabricated PSCS, including PDMS-rGO electrodes, P(VDF-TrFE) piezoseparator, and PVA/H_3_PO_4_ gel electrolyte, respectively. (**b**) Charging–discharging curves and (**c**) the short-circuit current curves of the PSCS device under different finger bending angles. Inset are optical pictures of the PSCS device stuck onto the finger. Reproduced from ref. [128], with permission. Copyright 2020, Chinese Materials Research Society; (**d**) schematic illustration of the synthesis of NiCoP/NiCoN heterostructure, the as-fabricated of P(VDF-TrFE)/BTO piezo-film, and the construction of SCSCP device; (**e**) self-charging process of SCSPC and corresponding discharge process at 3 µA. Reproduced from ref. [129], with permission. Copyright 2022, Elsevier; (**f**) self-charging profile of the fabricated piezo-supercapacitor under a compressive force of 30 N; (**g**) cycling performance of fabricated asymmetrical piezo-supercapacitor under compressive force. Reproduced from ref. [130], with permission. Copyright 2021, Elsevier.

Lu et al. [128] established a flexible Piezoelectric Self-charging Supercapacitor (PSCS) using a PDMS-Reduced Graphene Oxide (rGO))/C hybrid membrane as positive and negative symmetric electrodes, PVA/H_3_PO_4_ as a gel electrolyte, and a reticular P(VDF-TrFE) membrane as a piezoelectric separator (Figure 9a). The porous structure of reticulated P(VDF-TrFE) films fabricated by the electrospinning process is suitable for ion migration. When the finger is bent to 90°, the device is charged to 0.45 V in 17 s and discharged in 18.0 s with a stable charging current of 6.4 μA (Figure 9b,c).

Further, Gao et al. [129] designed a groundbreaking solid-state SCSPC featuring a NiCoP/NiCoN heterostructure as the positive electrode and active carbon (AC) as the negative electrode for energy storage. The separator used for generating a built-in piezoelectric field for energy harvesting was poly (vinylidene fluoride-co-trifluoroethylene)/barium titanate [P(VDF-TrFE)/BTO]. Figure 9d outlines the entire fabrication process, encompassing the synthesis of NiCoP/NiCoN heterostructures, P(VDF-TrFE)/BTO piezoelectric films, and SCSPC devices. The P(VDF-TrFE)/BTO piezoelectric films, equipped with piezoelectric properties, were created through spin-coating and polarization under a DC electric field. The NiCoP/NiCoN heterostructure, formed through in situ phosphating and nitriding, capitalizes on electronic structure optimization and strong heterointerface synergy to facilitate an efficient electron transfer and swift reaction kinetics. Functioning as a self-supporting electrode, the NiCoP/NiCoN heterostructure attains a high capacitance of 3544 mF cm^−2^ (1772 F g^−1^) and maintains exceptional cycling stability at a current density of 1 mA cm^−2^. Under a compressive force of 35 N, the device’s voltage ascends from 50 mV to 183 mV (133 mV charged) in just 146 s, as illustrated in Figure 9e.

3. Organic crystals

Verma et al. [130] constructed an asymmetric piezo-supercapacitor device. This device utilizes a cathode composed of a Nickel/Rochelle Salt (Ni/RS) NW array, an anode made of a Polypyrrole/Nickel/Rochelle Salt (PPy/Ni/RS) nanowire array, and Rochelle salt-based filter paper as the separator and energy harvester. The electrolyte is KOH. The device can achieve 700 mV self-charging within 15s under the action of a compression force of 30 N (Figure 9f). Under a compressive force of 10 N, the device exhibited self-charging characteristics for 8 s in the first cycle. Upon reapplying the mechanical force, the device demonstrated self-charging once more, confirming the repeatability of the developed piezoelectric supercapacitor (Figure 9g). The prepared Ni/RS//PPy/Ni/RS asymmetric device has an energy density of 166.23 Wh kg^−1^ at 0.24 KW kg^−1^.

#### 3.2.4. Flexible SCPSCs Based on Piezoelectric Ion (PI) Effect

**Figure 10:**
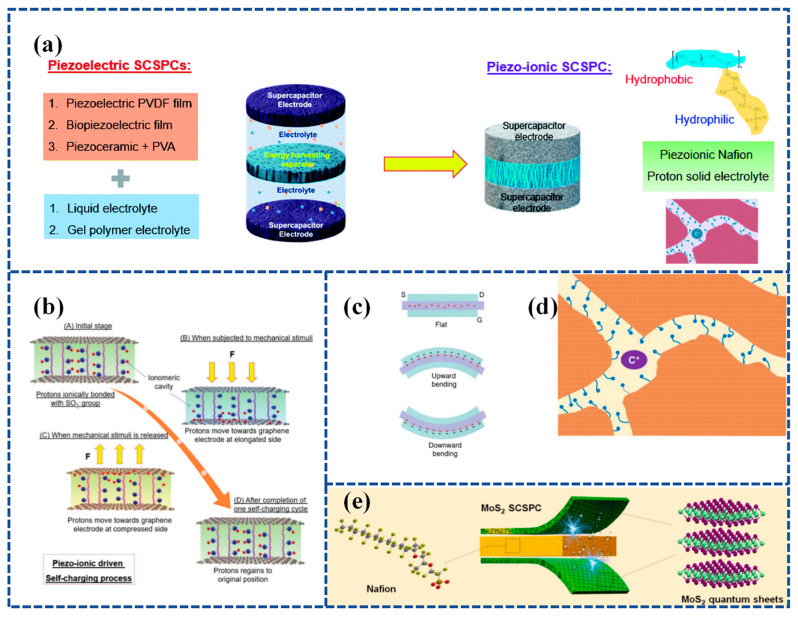
(**a**) The current architecture of SCSPC utilizing piezoelectric separators and the design of the novel piezo-ionic SCSPC in this work. The top and bottom of the right corner in Figure 10a show the molecular structure of Nafion and the ionomeric cavity present in Nafion; (**b**) mechanism of self-charging occurred in the Graphene PI-SCSPC; (**c**) proton movement in the Nafion film present in Graphene PI-SCSPC under the flat state, upward, and downward bending conditions. Reproduced from ref. [131], with permission. Copyright 2022, Royal Society of Chemistry; (**d**) schematic representation of the ionomeric channels present in Nafion for the free movement of cations (C^+^); (**e**) schematic representation of the fabrication of Nafion-based solid-state SCSPC using MoS_2_-QSs as supercapacitor electrodes. Reproduced from ref. [132], with permission. Copyright 2022, Elsevier.

SCSPCs have so far utilized two types of separators. The first type involves piezoelectric polymers and their copolymers or biopolymers [97,126], while the second type incorporates piezoelectric materials into PVA-based gel polymer electrolytes [118,119]. In the reported research, the operational principles of SCSPCs are explained based on the principles of piezoelectric electrochemistry. Recent research has focused on utilizing the piezoelectric ion properties of a polymeric electrolyte, specifically Nafion, as a separator and ion source for SCPSCs. Figure 10a illustrates the development trends of SCSPCs. Karthikeyan Krishnamoorthy et al. [131] fabricated an innovative SCSPC. This device incorporates a Nafion solid polyelectrolyte separator coated with graphene sheets to serve as a supercapacitor electrode. It demonstrates a self-charging process attributed to the piezoelectric ion (PI) effect. The self-charging process of Graphene PI-SCSPC is illustrated in Figure 10b. When the device is in an equilibrium state, free-moving protons combine with sulfate ions on Nafion. Upon external stimulation, protons move toward the elongated region existing on the graphene electrode, forming an electric double layer (EDL) on the surface of the graphene sheet. This process generates a Donnan potential through piezoelectric or mechanical ion effects. During this process, proton adsorption on the graphene surface leads to a net potential difference between the symmetrical graphene electrodes, thus completing the self-charging process. When the external mechanical stimulation is released, protons move from the graphene electrode back to the compressed region of the Nafion piezoelectric electrolyte membrane. Upon the complete cessation of external mechanical stimulation, the Graphene PI-SCSPC will return to its initial equilibrium state. The motion of protons within the Nafion membrane of the Graphene PI-SCSPC under flat, upward, and downward bending conditions is shown in Figure 10c. The Graphene PI-SCSPC can self-charge up to 341 mV under mechanical stimulation.

Compared to conventional liquid electrolytes, solid electrolytes in solid-state supercapacitors have the dual role of electrolyte and separator, and the use of solid electrolytes can effectively prevent short circuits between two electrodes and leakage of liquid electrolytes. The crucial feature of separators used in electrochemical systems is the presence of ion channels, where the movement of cations leads to the emergence of piezoelectric ion characteristics (Figure 10d). Further, Parthiban Pazhamalai et al. [132] exploited the piezo-ionic properties of Nafion (solid polyelectrolytes) and their bifunctional properties (separator and electrolyte) in SCSPC (using MoS_2_ quantum sheets as energy storage electrodes) and examined their self-charging performance. Experiments evaluating the piezo-ionic properties of Nafion polyelectrolytes show that they can generate peak voltages of 910 mV due to Donnan potential generation. Secondly, the electrochemical performance of solid-state MoS_2_-Nafion-MoS_2_ SCSPCs was examined, demonstrating their high capacity/capacitance (0.40 μAh cm^−2^/1.81 mF cm^−2^), energy density (252 μJ cm^−2^), and extended life cycle. Lastly, an exploration of the self-charging characteristics of MoS_2_-Nafion-MoS_2_ SCSPCs revealed their ability to self-charge up to 243 mV with excellent electromechanical stability. Furthermore, SCSPCs can be seamlessly integrated with wearable electronics. Figure 10e illustrates a schematic representation of the fabrication process for Nafion-based solid-state SCSPCs.

## 4. Current Challenges of Flexible SCSPCs

Although fast progress has been achieved, flexible SCSPCs still face many challenges and deficiencies.

1. Energy Density: One of the main challenges of flexible piezoelectric supercapacitors was their relatively low energy density compared to conventional batteries. Although they can store and release energy quickly, their total energy storage capacity per unit volume was lower than lithium-ion batteries or other traditional energy storage technologies.
2. Power Output: While piezoelectric supercapacitors excel in rapid energy release and high power output during piezoelectric deformation, they may not be reliable for applications requiring a continuous and sustained power supply.
3. Mechanical Durability: Flexible piezoelectric materials can suffer from mechanical fatigue over time, especially during repeated bending or stretching. This can lead to a decrease in the device’s overall performance and lifespan.
4. Charge/Discharge Cycles: The lifespan, that is, the number of charge/discharge cycles a piezoelectric supercapacitor can undergo without significant degradation, is another concern. Frequent and extensive cycling can lead to reduced capacitance and lower energy storage capabilities over time.
5. Integration Challenges: Integrating flexible piezoelectric supercapacitors into practical devices or systems may require further research and development to optimize their mechanical, electrical, and structural compatibility.
6. Energy Conversion Efficiency: The main disadvantage or limitation of SCSPC devices for practical applications may be the low efficiency of energy conversion and inadequate understanding of energy conversion and storage mechanisms.

It is worth noting that many of the above-mentioned issues have been addressed or mitigated through the development of new materials, design improvements, and manufacturing processes in ongoing research. As material engineering advances and new processing technology evolves, the performance and practicality of flexible piezoelectric supercapacitors are likely to develop further, making them more viable for a wider range of practical applications.

## 5. Conclusions and Perspectives

This review discusses the working principle, structural design, different piezoelectric electrodes, separators, and electrolyte materials used for supercapacitors with built-in PENGs. It can be concluded the development of built-in PENGs has significantly contributed to the advancement of sustainable and flexible supercapacitors, and these innovative devices have shown the potential to revolutionize energy storage and conversion technology by providing a clean, efficient, and sustainable solution to power wearable electronics, IoT devices, and other flexible applications. Meanwhile, the integration of various generators, including PENGs, with flexible supercapacitors will continue to be a major research focus in the field of energy harvesting and storage, and further research and development efforts should be directed toward improving the efficiency, scalability, and durability of these devices while exploring new materials and fabrication techniques to optimize their charging and powering performance. Additionally, the commercialization of these technologies requires the establishment of industry standards and regulations to ensure their safe and reliable operation. Ultimately, the success of built-in PENGs depends on their ability to deliver sustainability, efficiency, and adaptability. By continuing to push the boundaries of what is possible in this field, researchers and industry leaders can pave the way for a future where sustainable and flexible energy storage solutions are not only feasible but also widely accessible and affordable.

## Data Availability

Not applicable.

## Abbreviations

(ASCs)	Asymmetric Supercapacitors
(AFM)	Atomic Force Microscope
(AC)	Active Carbon
(BT)	Barium–Titanium
(CNFs)	Cellulose Nanofibers
(CC)	Carbon Cloth
(EDL)	Electric Double Layer
(EDLC)	Electric Double-Layer Capacitor
(GPE)	Gel Polymer Electrolyte
(LIB)	Lithium-ion Batteries
(NW)	Nanowire
(NP)	Nanoparticles
(NC)	Nanocomposite
(PPy)	Polypyrrole
(PTA)	Phosphotungstic Acid
(PZT)	Lead Zirconate Titanate
(PVA)	Polyvinyl Alcohol
(PVDF)	Poly(vinylidene fluoride)
P(VDF-TrFE)	Poly(vinylidene fluoride-co-trifluoroethylene)
(PENG)	Piezoelectric Nanogenerator
(PSCS)	Piezoelectric Self-charging Supercapacitor
(PSCFS)	Piezoelectric Self-charging Flexible Supercapacitor
(rGO)	Reduced Graphene Oxide
(SC)	Supercapacitor
(SCAPSC)	Self-charging Asymmetric Piezo-electric Supercapacitor
(SCSC)	Self-chargeable Supercapacitor
(SCHSC)	Self-charging Hybrid Supercapacitor
(SCSPC)	Self-charging Supercapacitor Power Cell
(SCPSC)	Self-charging Piezoelectric Supercapacitor
(SCHSD)	Self-charging Hybrid Supercapacitor Device
(SPSSC)	Self-powered Symmetrical Supercapacitor
(XRD)	X-ray Diffraction
